# Qualitative evaluation of a molecular point-of-care testing study for influenza in UK primary care

**DOI:** 10.3399/BJGPO.2024.0112

**Published:** 2024-11-13

**Authors:** Charis Xuan Xie, Uy Hoang, Jessica Smylie, Carole Aspden, Elizabeth Button, Cecilia Okusi, Rachel Byford, Filipa Ferreira, Sneha Anand, Utkarsh Agrawal, Matthew Inada-Kim, Tristan Clark, Simon de Lusignan

**Affiliations:** 1 Nuffield Department of Primary Care Health Sciences, University of Oxford, Oxford, UK; 2 Wolfson Institute of Population Health, Barts and The London School of Medicine and Dentistry, Queen Mary University of London, London, UK; 3 Royal Hampshire County Hospital, Hampshire Hospitals Foundation Trust, Winchester, UK; 4 School of Clinical and Experimental Sciences, Faculty of Medicine, University of Southampton, Southampton, UK; 5 University Hospital Southampton NHS Foundation Trust, Southampton, UK

**Keywords:** point-of-care testing, general practice, influenza, human, primary healthcare, general practitioners

## Abstract

**Background:**

Influenza contributes to the surge in winter infections and the consequent winter pressures on the health service. Molecular point-of-care testing (POCT) for influenza may improve patient management by providing rapid and accurate clinical diagnosis to inform the timely initiation of antiviral therapy and reduce unnecessary admissions and antibiotics use.

**Aim:**

To explore factors that influence the adoption or non-adoption of POCT in English general practices and provide insights to enable its integration into routine practice workflows.

**Design & setting:**

A qualitative implementation evaluation was conducted in 10 general practices within the English national sentinel network (Oxford RCGP Research and Surveillance Centre), from April–July 2023.

**Method:**

Using the Non-adoption, Abandonment, Scale-up, Spread, and Sustainability (NASSS) framework, data collection and analysis were conducted across 10 practices. We made ethnographic observations of the POCT workflow and surveyed the practice staff for their perspectives on POCT implementation. Data were analysed using a mix of descriptive statistics, graphical modelling techniques, and framework analysis.

**Results:**

Ethnographic observations identified the following two modes of POCT integration into practice workflow: (1) clinician POCT workflow, which typically involved batch testing owing to time constraints; and (2) research nurse or healthcare assistant POCT workflow, which was characterised by immediate testing of individual patients. Survey data indicated that most primary care staff considered the POCT training offered was sufficient and these practices were ready for change. Some participants agreed that there was the capacity and resources to integrate POCT into workflows. It was uncertain as to whether POCT required changes to organisational routines and processes.

**Conclusion:**

General practices should demonstrate flexibility in the workflow and workforce they deploy to integrate POCT into routine clinical workflow.

## How this fits in

There are limited qualitative evaluations on the implementation of point-of-care testing (POCT) for influenza in primary care settings. However, POCT for influenza may help reduce winter pressure in general practices (and potentially in secondary care) by improving patient management with rapid and accurate clinical diagnoses that inform timely decision making, including the prescription of antivirals and enhanced antimicrobial stewardship. This study used ethnographic observations and a semi-structured questionnaire to explore the factors impacting POCT adoption in 10 practices within the English national sentinel surveillance network. Our findings identified that practices used one of two models to incorporate POCT into clinical workflow: (1) clinician-led POCT sampling, typically conducted post-clinical consultation, and involving batch testing owing to time constraints; and (2) research nurse or healthcare assistant-led POCT sampling, characterised by immediate testing of individual patients. Most primary care staff perceived POCT implementation as a simple procedure; however, whether and how POCT can be integrated into staff routine workload and clinical pathway requires further research.

## Introduction

The seasonal surge in acute respiratory infections (ARI), including influenza, contributes to significant winter pressures on health services every year.^1,2^ Annual influenza vaccination is recommended; however, its effectiveness varies depending on the patient group vaccinated and the match between the vaccine and circulating strains.^3^ Antiviral treatment of influenza can be effective when started shortly following symptom onset and is recommended by national guidelines^4^ for high-risk individuals. However, the initiation of appropriate antiviral drugs remains challenging,^5^ and the frequency of prescribing antiviral medications to high-risk patients in primary care is low.^6^

In the UK, the diagnosis of influenza in primary care typically relies on clinical assessment, where decisions are guided by the presentation of symptoms and local prevalence data. Additional testing methods for more accurate diagnoses include molecular diagnostic tests, such as reverse transcription-polymerase chain reaction. During the COVID-19 pandemic, lateral flow assays were extensively employed for rapid coronavirus testing; similar rapid antigen tests are also available for influenza.^7^

Point-of-care testing (POCT), diagnostic testing performed at or near the site of patient care as opposed to testing using conventional centralised laboratories,^8^ has emerged as a potentially useful tool in this regard. There have been recent significant advancements in the development and use of rapid molecular POCT platforms for influenza, which have been shown to improve antiviral prescribing in hospital settings.^9–11^ The use of POCT has also been associated with other benefits in secondary care including reducing the length of stay, improving infection control, and reducing the overall cost of hospitalisation.^12,13^

In 2018, English national guidelines were published for implementing rapid POCT for seasonal influenza and other respiratory viruses.^14^ Key factors influencing successful implementation were identified as the type of POCT platform, clinical pathways and staff training, clinical governance, cost, and monitoring of effectiveness.^14^

Following the national recommendations, we have conducted a series of POCT studies within the English national sentinel network managed by the Oxford Royal College of General Practitioners (RCGP) Research and Surveillance Centre (RSC). In 2020, we undertook a mixed-method feasibility study on influenza POCT across six practices, which demonstrated that it is feasible to implement POCT in general practices and it may improve antiviral use and reduce unnecessary antibiotic use.^15^

We conducted 'The Impact of Point-of-Care Testing for Influenza on Antimicrobial Stewardship (PIAMS)',^16^ within which this qualitative investigation was carried out to understand the factors influencing the adoption or non-adoption of POCT in general practices. The insights drawn from this study could inform the effective integration of POCT into clinical workflow, particularly to prepare for managing the winter pressures associated with respiratory infections.

## Method

### Study design

This is a qualitative study nested within the PIAMS POCT implementation study. We used the Non-adoption, Abandonment, Scale-up, Spread, and Sustainability (NASSS) framework^17^ to guide the evaluation of POCT delivery in primary care settings. We undertook ethnographic observations of the POCT process as it is deemed a preferred methodological approach for studying health technologies within 'complex social systems'.^18^ Additionally, we surveyed the practice staff for their perspectives on POCT implementation using a semi-structured questionnaire adapted from the NASSS framework. This study follows the Standards for Reporting Qualitative Research reporting guideline.^19^

### NASSS theoretical framework

The NASSS framework^17^ consists of the following seven domains: the condition or illness; the technology; the value proposition; the adopter system; the organisation(s); the wider (institutional and societal) context; and the interaction and mutual adaptation between all these domains over time. This evidence-based theory is specifically crafted for studying health technology implementation and provides a sociotechnical perspective to inform future POCT implementation and scale-up within primary care. Since we were primarily interested in how human-technology interaction impacted routine workflow in the context of primary care practice, and practices were not required to purchase POCT machines or equipment as part of this study, our ability to look at the value proposition for POCT adoption was thus limited. We therefore adopted three domains of the NASSS framework (that is, the technology, the intended adopters, and the organisation) to assess the phenomenon.

### The intended adopter: primary care staff

The POCT adopters in our study are practice staff who implement and deliver the influenza POCT service. They may include clinicians (for example, GPs or nurse practitioners), research nurses, healthcare assistants (HCAs), practice managers, and administrators. Practice staff received hands-on training on administering the test, provided face-to-face or virtually by practice liaison staff from the RCGP RSC. This consisted of the following four components: (1) study overview; (2) information about the cobas Liat analyser; (3) information about the flu or respiratory syncytial virus (RSV) test; (4) how to run the rapid test and record the results (see Supplementary Table S1). They were also given instructional leaflets, with access to a helpline during office hours should they require further information on assessing patients with influenza-like illness, consenting them for the study, and using POCT to detect respiratory viruses. Calibration of each machine was performed automatically before each test. When the patient attended the practice, a nasopharyngeal swab was collected from consented eligible patients by a trained practitioner, inserted in a test kit, and tested on the cobas Liat analyser to detect influenza. Practice staff were asked to code that the patient had a POCT swab and the results of the test to facilitate identification of POCT tests in the computerised medical record (CMR).

### The technology: point-of-care testing

The POCT, the technology we implemented, was the cobas Liat system. It is an integrated diagnostic solution that comprises the automated cobas Liat analyser and the cobas Liat assay tubes (test kit) for in vitro testing. It is a portable POCT machine that performs real-time polymerase chain reaction (PCR) tests for influenza A/B, RSV, and SARS-CoV-2 with a time to result of around 20 minutes.^20^ The machine and test kits have Conformité Européenne mark and US Food and Drug Administration approval for rapid influenza testing and have been shown to have high sensitivity and specificity for detecting influenza.^20–24^ This study did not examine the accuracy of the POCT machine but used the machine for its approved purpose.

### The organisation: general practices

UK general practice is a registration-based system (patients are registered with a single GP), and it has used CMR systems since the 1990s. General practices within the Oxford RCGP RSC network are continually provided with feedback on their CMR coding. The resulting data quality within the database means it provides a world-leading dataset for health services research. In the PIAMS study, we aimed to recruit practices within the Oxford RCGP RSC sentinel network with the capacity to undertake POCT testing and that had previously been involved in SARS-CoV-2 POCT through the RAPid community Testing fOR COVID-19 (RAPTOR-C19) study.^25^ Those practices with a history of less than 80% complete data returns during the previous winter season were excluded, resulting in 10 participating practices being recruited. The age–sex profile of study practices is shown in Supplementary Figure S1. Other practice characteristics, including practice size, ethnicity, and socioeconomic status of the practice population, measured by the Index of Multiple Deprivation (IMD), are depicted in Table 1. IMD is a nationally available measure of socioeconomic status assigned based on postcode.^26^ Study practices were offered a one-off payment at the start of the study, for their attendance at study training events and site initiation visits. They were also offered a payment once the study had been completed. In addition, to encourage patient sampling, they were offered financial incentives for each patient recruited and to encourage data recording they were offered a payment for sending data to the research team.

**Table 1.** PIAMS study practice characteristics

Characteristics of practice	A	B	C	D	E	F	G	H	I	J
Practice size (registered patients)	18 128	15 672	20 553	19 125	7305	18 324	10 541	8909	9434	16 435
Ethnicity (%)^a^										
White	96.8	96.2	94.9	30.6	49.2	12.0	53.8	60.0	49.2	54.7
Mixed or multiple ethnic groups	1.3	1.5	1.6	6.7	5.4	2.6	6.7	4.9	5.4	5.8
Asian or Asian British	1.5	1.6	2.3	34.8	27.4	73.9	16.8	24.3	27.4	13.9
Black, African, Caribbean, Black British	0.3	0.3	0.9	25.4	14.1	9.5	17.1	7.8	14.1	22.1
Other ethnic group	0.1	0.4	0.4	2.5	3.9	2.1	5.6	3.0	3.9	3.4
IMD decile^b^	10	10	8	5	2	4	1	3	2	2

a Ethnic group statistics from the 2011 Census for England and Wales at postcode sector level. ^b^IMD decile: from one the most deprived 10% geographic area to 10 the least deprived 10% of geographic area.

IMD = Index of Multiple Deprivation. PIAMS = Impact of Point-of-Care Testing for Influenza on Antimicrobial Stewardship study.

### Ethnographic observation

We used an opportunistic sampling method to select practices for the ethnographic observation. All practices were contacted by the PIAMS study team (via email) to confirm their availability to host a researcher for the process observation. The fieldwork was undertaken in May 2023, several months after the practices had started POCT sampling. This timing was deliberately chosen to allow the practices to integrate the new testing procedures into workflow and to ensure that practice staff had accumulated experience with the POCT, including its strengths and limitations. A trained member (CX) of our research team, specialising in implementation science, observed the interaction between the machine operator and patient during the POCT swabbing. However, owing to a low flu rate in May, actual swabbing observations were sporadic. In instances without swabbing, the machine operator demonstrated the procedure for the researcher, simulating a patient’s presence. Ad hoc informal interviews with the practice staff also occurred during the observation, and the discussion was mainly around the use of technology, the operating procedures, and how the POCT impacts the original workflow. Field memos were taken during the observation periods with the following information collected:

- process duration including starting point and ending point;
- waiting time;
- machine operator;
- physical location of the POCT machine;
- how POCT sampling to detect respiratory viruses is undertaken;
- how the POCT machine is operated (that is, what actions are performed);
- other technologies involved in the process;
- other events involved in the process;
- how easy the POCT machine is to use;
- changes to the staff’s routine workflow; and
- challenges when using POCT.

### Semi-structured questionnaire

We used purposive sampling to recruit practice staff who were involved in the POCT implementation to complete the questionnaire. They included GPs, nurses, practice managers, HCAs, and research administrators. Participants from three Oxford practices were sent individual invitation emails containing the link to the questionnaire, while the rest received invitations via the single point of contact for research liaison at their practice. The semi-structured questionnaire was live from June to July 2023, with reminders sent each week.

The questionnaire was created on the Joint Information Systems Committee (Jisc) survey platform. The questions were adapted based on three domains of the NASSS complexity assessment toolkit (NASSS-CAT), that is the technology, the intended adopters; and the organisation.^27^ For each question, we employed a five-point Likert scale for responses, ranging from 'strongly disagree' to 'strongly agree'. To ensure diverse perspectives, both positively and negatively worded questions were incorporated. An open text box was also provided at the end of each question to allow for further comments or elaboration.

### Data analysis

While our study is primarily qualitative, we employed descriptive statistics to provide a clear overview of the data collected from surveys, facilitating a clearer contextualisation of the qualitative findings. Framework analysis, informed by the NASSS theory, was conducted to group the individual questions. Negatively phrased items were reverse-coded to align with the positive direction of the group theme. To facilitate process analysis from ethnographic observation, we employed a graphic notation technique — Business Process Modelling Notation (BPMN) — to visualise the POCT swabbing workflow. Alongside this, we also developed Unified Modelling Language (UML) use-case diagrams to capture high-level user requirements when using POCT. Both BPMN and UML use-case diagrams were chosen owing to their distinct and complementary strengths in understanding and representing complex processes. BPMN is specifically designed to provide a standardised, intuitive representation of business process,^28^ making it ideal for capturing the sequence of the POCT swabbing workflow in the domain of organisation. On the other hand, UML use-case diagrams that emphasise complete transactions viewed from the user perspective,^29^ which could help us clarify the expectations of users (for example, the technology adopters) when interacting with POCT. Together, these techniques allowed us to gain a comprehensive insight into both the procedural aspects and the user-focused dimensions of POCT, ensuring that our analysis was holistic and well-rounded.

## Results

Through the lens of the NASSS framework, our investigation sheds light on the various elements crucial to the adoption and non-adoption of POCT within primary care settings (see Table 2).

**Table 2.** Results summary according to the NASSS domains

NASSS domain	Description
The condition	Focusing on acute respiratory viruses. Our study did not extend to the comparison of POCT adoption for other conditions.
The technology	The usability of the POCT machines was identified as a pivotal factor for technology adoption.
The value proposition	The practices were not required to purchase POCT machines or equipment as part of this study, thus limiting our ability to look at the value proposition for POCT adoption.
The intended adopter	Our results underscored that the roles and responsibilities of the machine operators emerged as a vital element in adopting POCT within a primary care setting.
The organisation	Organisational-wide support for change and collective buy-in were considered essential in POCT implementation.
The wider system	The study was nested within the English sentinel surveillance network and supported by a study team experienced in delivering infectious disease surveillance; however, we are not able to comment on the adoption of POCT across general practice more specifically where there may not be support and ongoing funding for rapid primary care diagnosis.
Embedding and adaptation over time	The study was performed over one winter season and thus we are not able to comment on the sustained adoption of POCT in primary care over time.

NASSS = Non-adoption, Abandonment, Scale-up, Spread, and Sustainability. POCT = point-of-care testing

### Ethnographic observation

**Figure 1.**
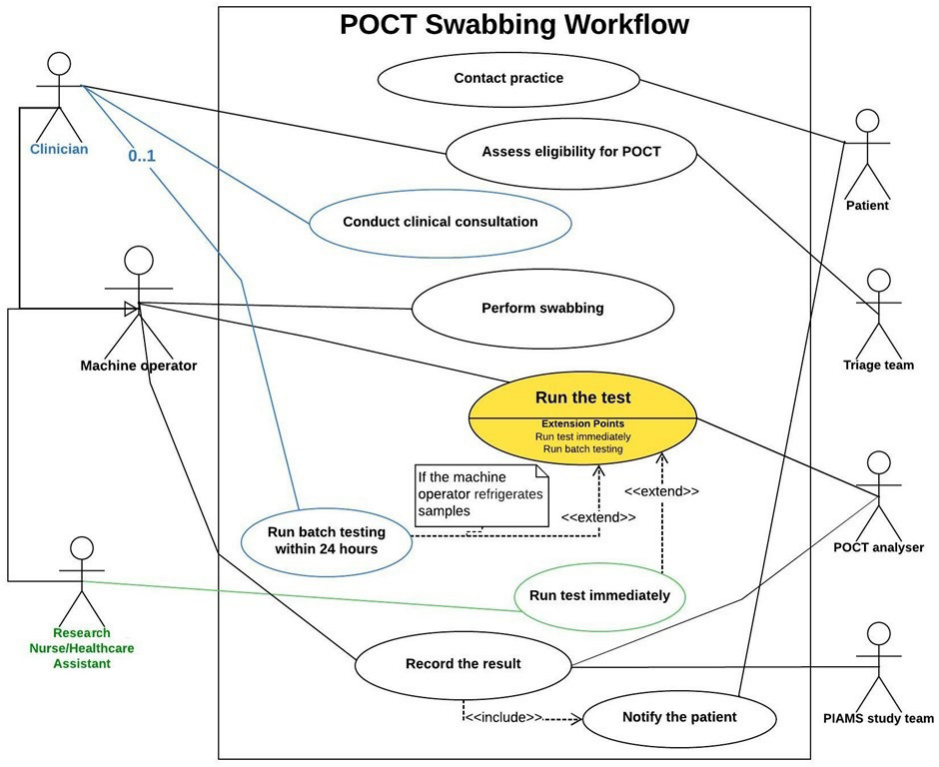
Unified Modelling Language (UML) use-case diagram for point-of-care testing (POCT) swabbing process. Footnotes: Rectangle: represents the boundary of the POCT process, encapsulating all related use-cases within. Ellipse: denotes a 'use-case', illustrating a specific function or task within the process. Stick figure: symbolises an 'actor', indicating an external entity, whether human, technology, or a role that interacts with the process. Lines: drawn between actors and use cases, these lines depict the relationships, indicating which actor can initiate or is involved in which use case.<< extend >> Relationship: denotes optional, conditional behaviour that can extend the basic functionality of a use-case.<< include >> Relationship: represents a use-case that is always included within another use-case, meaning it is a compulsory part of the base use-case’s process. Multiplicity: this is indicated on the relationship lines, showing the number of times an actor interacts with a use-case or vice versa. For instance, '0..1' means that not all clinicians run batch testing.

We conducted ethnographic observations in four general practices and collected the data outlined in Table 3. Drawing from the fieldwork data, we created a flowchart (Supplementary Figure S2) to provide a high-level representation of the POCT workflow. Additionally, we developed a UML use-case to showcase the interactions between adopters and the technology (Figure 1) and used BPMNs to detail the process further (see Supplementary Figures S3 and S4 for details).

**Table 3.** Information gathered from ethnographic observation

Site visited	Machine operator	Other roles involved in POCT delivery?	Machine location	Main POCT workflow	Process duration (inc. waiting time)	Other technologies involved in the process?	Other events involved in the process?
Practice A	Emergency care practitioner	None	A communal space between two consultation rooms	See Figure 1 highlighted in blue	Swabbing: 5 minutes; running tests in the machine within a blocked time period (average 1 hour/day)	CMR	None
Practice B	Research nurse or GP	GPs, triage team	Storage room	See Figure 1	25 minutes	CMR	Clinical consultation
Practice C	Research nurse	GPs, triage team	Clean utility room	See Figure 1 highlighted in green	25 minutes	CMR	None
Practice D	Healthcare assistant	Everyone in the practice	Triage room	See Figure 1 highlighted in green	25 minutes	CMR	None

CMR = computerised medical record. Inc = including. POCT = point-of-care testing

The UML use-case diagram delineates the overarching user interactions associated with the POCT process. The general workflow started with a patient contacting the practice for an appointment, followed by the triage team assessing their eligibility for POCT. Then the eligible patient would be referred to the machine operator for swabbing and testing. There were two types of machine operators who led different POCT workflow: (1) the clinician (for example, GPs) workflow; and (2) the research nurse or HCA workflow. Distinct use-cases for these two roles are highlighted in blue (for clinicians) and green (for research nurses and HCAs). A notable difference emerged in the clinicians' workflow, where they undertake an additional step of conducting clinical consultations before swabbing, a process absent in the research nurses' flow. Another divergence appears in the test execution phase (highlighted in yellow): some clinicians, owing to time constraints, opted to refrigerate the samples and batch-test them within a 24-hour window, while research nurses and HCAs typically conduct the test immediately post-swabbing as they had assigned specific times for operating the POCT. Such variances underscore how the introduction of POCT has influenced the regular workflow of clinicians in primary care settings.

### Semi-structured questionnaire

We received 11 survey responses from seven out of 10 practices, involving GPs (*n* = 3), nurses (one research nurse and one nurse practitioner), practice managers (*n* = *2*), administrators (*n* = 2), HCA (*n* = 1), pharmacists (one research pharmacist and one GP pharmacist). Some participants have more than one role. Regarding POCT use, 63.6% (*n* = 7) of participants are frequent POCT users (more than five times), while the rest (*n* = 4) used POCT less than five times.

**Figure 2.**
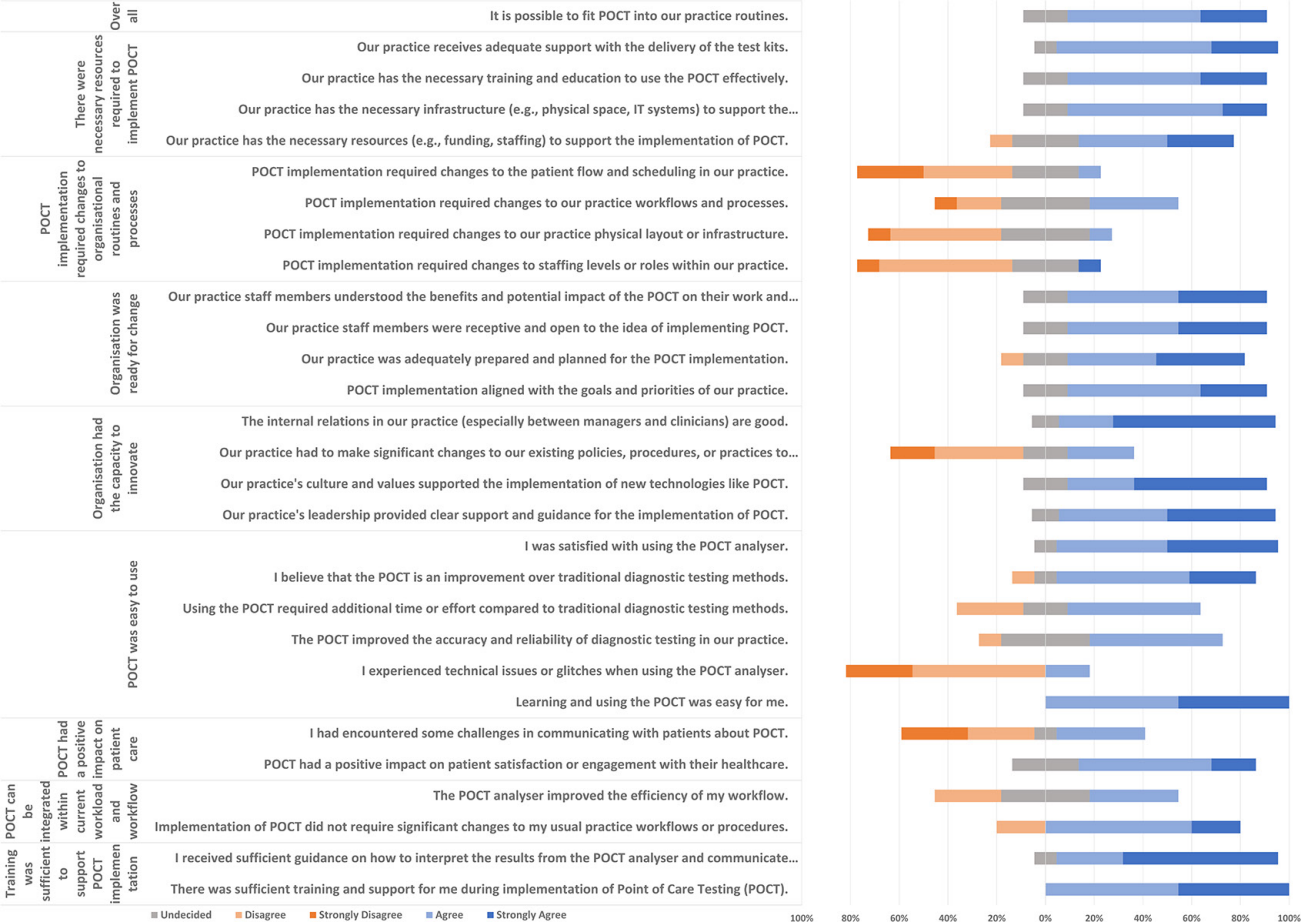
Results from individual questions. POCT = point-of-care testing

Table 4 presents the synthesised findings from the questionnaire. Results from individual questions were provided in Figure 2. Overall, implementing POCT in primary care was largely perceived as a simple procedure (Supplementary Figure S5).

#### The intended adopter

In general, the majority of primary care staff expressed strong agreement about the sufficiency of training supporting POCT implementation, with a mean of 4.50 (Table 4). However, participants’ perspectives varied on whether POCT can be integrated as part of staff routine as the potential of POCT in improving the efficiency of clinical workflow remained questionable (Figure 2). In addition, it is uncertain whether POCT would have a positive impact on patient care, largely owing to some practice staff encountering challenges in communicating with patients about the POCT (Figure 2).

**Table 4.** Descriptive summary of the 5-point Likert scale survey responses

NASSS domain	Factors impacted POCT implementation	Strongly disagree	Disagree	Undecided	Agree	Strongly agree	**Mean** ^**a**^	Mode
*The intended adopter*	Training was sufficient to support POCT implementation	0	0	1	9	12	4.50	Strongly agree
	POCT can be integrated within current workload and workflow	0	5	5	10	2	3.41	Agree
	POCT had a positive impact on patient care	0	4	4	9	5	3.68	Agree
*The technology*	POCT was easy to use	0	10	8	32	16	3.82	Agree
*The organisation*	Organisation had the capacity to innovate	0	3	8	13	18	4.10	Strongly agree
	Organisation was ready for change	0	1	8	20	15	4.11	Strongly agree
	POCT implementation required changes to organisational routines and processes	6	17	14	6	1	2.52	Disagree
	There were necessary resources required to implement POCT	0	1	10	30	14	4.04	Agree

^a^Weighted mean, weight assigned as: strongly disagree: 1, disagree: 2, undecided: 3, agree: 4, strongly agree: 5.

NASSS = Non-adoption, Abandonment, Scale-up, Spread, and Sustainability. POCT = point-of-care testing

#### The technology

Although nearly all participants were satisfied with using the POCT analyser, negative ratings were seen in the aspect of additional time required to process samples and concerns about whether POCT could improve the accuracy and reliability of diagnostic testing (Figure 2). This varied feedback was also reflected by the open-text comments addressing the time-intensive sample processing (quotes 1 and 2) and the predominance of negative virus detection results (quote 3):

Quote 1: *'It does take some time to process the sample and then await the results.'*

Quote 2: *'Length of time to run the sample can be time-consuming.'*

Quote 3: *'Nearly all our results were negative for virus detection. If there were more positives then patient engagement with the results would be higher.'*

#### The organisation

There was substantial agreement on the factors of the organisation’s innovative capacity (mean = 4.10) and readiness for change (mean = 4.11). Some participants agreed that there were necessary resources available for POCT implementation (mean = 4.04). However, it is uncertain as to whether POCT required changes to organisational routines and processes (mean = 2.52), which resonates with the findings from the ethnographic observations.

## Discussion

### Summary

This qualitative study conducted in primary care practices explored three NASSS domains (the technology, the adopters, and the organisation) that impacted POCT implementation.

From ethnographic observations, we found that there were two main ways that POCT was integrated into practice workflow: (1) clinician-led POCT swabbing, typically conducted post-clinical consultations and involving batch testing owing to time constraints; (2) research nurse or HCA-led POCT swabbing, characterised by immediate testing of individual patients.

Additionally, survey responses highlighted that most primary care staff considered there was sufficient training for supporting POCT implementation, and the practices had the capacity to innovate, possessed the necessary resources to adopt POCT, and were ready for the change. However, some concerns were raised about the integration of POCT into routine staff workload, its potential to improve workflow efficiency, and the need for changes to clinical pathways in general practices. Despite its relatively quick 20-minute turnaround time, some staff still perceived this as a barrier in the busy clinic setting.

### Strengths and limitations

This study provided in-depth insights into the experiences and perspectives of participating primary care staff in the POCT implementation. Through direct observations and complementary questionnaires, we were able to understand the context and intricacies of POCT implementation within various practice settings.

However, the study’s short duration and small sample size, and the seasonal nature of respiratory infections might limit the generalisability of our findings. And it is important to note that this study was conducted during a period of low disease prevalence, which might underestimate the challenges during peak times. We recognise the presence of selection bias, as the practices involved were selected from the RCGP RSC and were all English practices, and likely to be more research active. To mitigate this, we ensured that a diverse range of practice types within the network were included, such as varying practice sizes, diverse ethnicity groups, and different levels of deprivation. Additionally, as this evaluation was conducted in the late implementation phase, there might be recall bias that could influence participants' accuracy in recalling their experiences with POCT. To address this, we triangulated the data and used consistent prompts derived from the NASSS framework during observations and online surveys to help participants recall specific instances rather than general impressions.

Furthermore, this implementation evaluation primarily focused on the technology users (that is, primary care staff) within general practices and their interactions with POCT. As a result, we may have overlooked other domains that could influence POCT adoption, including the disease, the technology’s value proposition, and the broader social context, as highlighted in the NASSS theory. Nevertheless, the framework remains highly relevant and was instrumental in guiding our data collection and analysis, providing a structured approach that enhanced the depth and reliability of our findings. While our study provided comprehensive insights into the factors influencing POCT implementation, it did not seek to hierarchically rank the influencing factors by their significance. Instead, our focus was on capturing the multifaceted nature of the issue.

Also, our focus was on a single POCT-test platform, and our findings might not fully represent other platforms that could have different advantages or disadvantages. Some participants are non-clinical staff and may use POCT only infrequently, their experiences and perceptions still enrich our understanding of the POCT adoption challenges and opportunities across various roles and frequencies of use. Questionnaires were initially sent to three targeted Oxford practices and subsequently to seven more practices via a central contact. This approach widened our sample but limited our ability to precisely measure the response rate, which may affect the representativeness of our findings.

### Comparison with existing literature

While there are limited qualitative evaluations focusing on the implementation of POCT for influenza in the primary care landscape,^30^ our study’s findings echo some key themes observed in past research. Specifically, our conclusions regarding implementation factors are consistent with the previous feasibility study that assessed the introduction of flu POCT across six general practices in the UK.^15^ This earlier work similarly identified machine operator characteristics and the clinical pathways to testing as significantly influencing the POCT rollout. Another UK-based primary care study^31^ examined the practice staff views on the use of point-of-care C-reactive protein testing, suggesting the major barriers to adoption were cost, time, machine accessibility, and workflow impact. They also found that more frequent POCT users typically had a dedicated staff member and the machine conveniently placed in their consultation room, which resonates with patterns we have also observed. One US study evaluated influenza POCT service in the community pharmacy setting,^32^ concluding that it was feasible and addressing the importance of increasing patient and provider awareness of POCT, pharmacist acceptance, and leadership support to facilitate successful implementation. Finally, a systematic review that broadened its lens to encompass various types of POCT applications in primary care reported four salient determinants: turnaround time, technical efficacy, positive predictive value, and negative predictive value,^30^ which is reinforced in our results.

### Implications for research and practice

Our findings provide insights to inform future effective POCT implementation in primary care, including newly formed ARI hubs. In response to the increasing winter pressure, NHS England funded 363 ARI hubs that were rapidly set up to increase community capacity for managing ARI.^2^ By March 2023, these hubs had seen 730 000 patients and are likely to be required every year going forward. These ARI hubs represent an excellent opportunity to test the implementation of POCT for influenza and other respiratory pathogens using standardised protocols and pathways and assess their large-scale impact.

During the observation, some practice staff were particularly enthusiastic about promoting and facilitating POCT adoption. These individuals were the implementation 'champions',^33^ who drove the successful implementation of health information technology.^34,35^ Proactive identification and engagement with these champions is likely to help overcome implementation challenges.

In general, the influenza POCT used in the study was considered to be a user-friendly tool, however, it was uncertain if it improved workflow and patient care. Future studies of POCT are needed to confirm clinical efficacy and efficiency.

Incorporation of POCT into workflow required changes in workflow and staff had to employ adaptive strategies. We predominantly saw two strategies, batch testing by GPs and multiprofessional integration into workflow. While batch testing can be more compatible with busy workflows, it may compromise the intended quick turnaround of POCT, especially crucial during peak influenza seasons.

Organisational culture also had a pivotal role in successful implementation. Training sessions could also include strategic resource allocation and foster an implementation culture to support effective uptake.^36^

One practice showed high sampling rates, driven primarily by pervasive organisational support. Every individual, irrespective of their role, engaged with the POCT study and directed eligible patients towards it. Beyond leadership, a unified organisational commitment was crucial for effective implementation. General practices and ARI hubs should aim for both informed leadership and collective buy-in across the organisation.

Our investigation into the adoption of POCT within UK general practices provides significant insights into practical implementation factors, which are often overlooked in POCT research.^30^ Understanding these factors is crucial for the successful adoption of POCT across diverse healthcare systems, particularly in settings where primary care is delivered by multidisciplinary teams and where there is specific funding support for POCT.

In conclusion, this qualitative work within the PIAMS study highlights the multifaceted nature of implementing POCT in primary care settings, emphasising the need for easy-to-use technology, sufficient staff time for training and testing, and greater organisational preparedness. Our findings suggest the importance of a flexible, adaptable implementation strategy to accommodate practice variations and staff needs. For example, when clinicians are short of time, employing a POCT batch-testing approach or assigning a specialised role (for example, research nurse) to operate POCT could help them navigate their workload. To validate and expand on our findings, future research should consider larger and longer-duration studies, explore a broader range of POCT platforms, including lateral flow devices (LFDs), and conduct cost-effectiveness studies to fully understand optimal strategies and conditions for successful POCT integration in diverse primary care settings and ARI hubs.

## Data

The data that support the findings of this study are available from Oxford-Royal College of General Practitioners (RCGP) Research and Surveillance Centre (RSC) but restrictions apply to the availability of these data, which were used under licence for the current study, and so are not publicly available. Data are, however, available from the authors upon reasonable request and with permission of RCGP RSC.
